# Antimicrobial Peptides and Cell-Penetrating Peptides for Treating Intracellular Bacterial Infections

**DOI:** 10.3389/fcimb.2020.612931

**Published:** 2021-02-05

**Authors:** Danieli F. Buccini, Marlon H. Cardoso, Octavio L. Franco

**Affiliations:** ^1^ S-inova Biotech, Programa de Pós-Graduação em Biotecnologia, Universidade Católica Dom Bosco, Campo Grande, Brazil; ^2^ Centro de Análises Proteômicas e Bioquímicas, Pós-Graduação em Ciências Genômicas e Biotecnologia, Universidade Católica de Brasília, Brasília, Brazil

**Keywords:** intracellular bacteria, antimicrobial peptides, cell-penetrating peptides, AMPs, CPPs

## Abstract

Bacterial infections caused by intracellular pathogens are difficult to control. Conventional antibiotic therapies are often ineffective, as high doses are needed to increase the number of antibiotics that will cross the host cell membrane to act on the intracellular bacterium. Moreover, higher doses of antibiotics may lead to elevated severe toxic effects against host cells. In this context, antimicrobial peptides (AMPs) and cell-penetrating peptides (CPPs) have shown great potential to treat such infections by acting directly on the intracellular pathogenic bacterium or performing the delivery of cargos with antibacterial activities. Therefore, in this mini-review, we cover the main AMPs and CPPs described to date, aiming at intracellular bacterial infection treatment. Moreover, we discuss some of the proposed mechanisms of action for these peptide classes and their conjugation with other antimicrobials.

## Introduction

Over the years, bacteria have been adapting to survive, become resistant to drugs, and make the host’s phagocytes their home (Cornejo et al., 2017). Intracellular bacteria have received special attention since they are extremely challenging to detect and treat (Mitchell et al., 2016). Once internalized, they can remain inactive but persist, causing recurrent infections and chronic illnesses (Monack et al., 2004). Many of the treatments currently used for internalized bacteria are ineffective at therapeutical doses (Monack et al., 2004). For example, conventional antibiotics used in treatments against extracellular bacteria have reduced permeability in intracellular bacterial infections (Proctor et al., 2006; Lehar et al., 2015).

Although antibiotics can kill the internalized bacteria, these microorganisms have shown different resistance profiles, thus often rendering the treatment ineffective (Proctor et al., 2006; Lehar et al., 2015). In this current scenario, the search for effective alternative ways of killing intracellular bacteria is imperative. Promising alternatives are antimicrobial peptides (AMPs) and cell-penetrating peptides (CPPs) for direct antibacterial activities or for the delivery of antibacterial cargos and peptide nucleic acids (PNAs) within the host cell (Splith and Neundorf, 2011; Le et al., 2017).

Antimicrobial peptides (AMPs) are commonly found in nature, also called natural antibiotics (Felício et al., 2017). These molecules play a critical role in the first line of defense against pathogens, being expressed constitutively or induced by different types of cells in response to infectious or inflammatory stimuli (Kumar et al., 2018; Yount et al., 2019).

**Figure 1:**
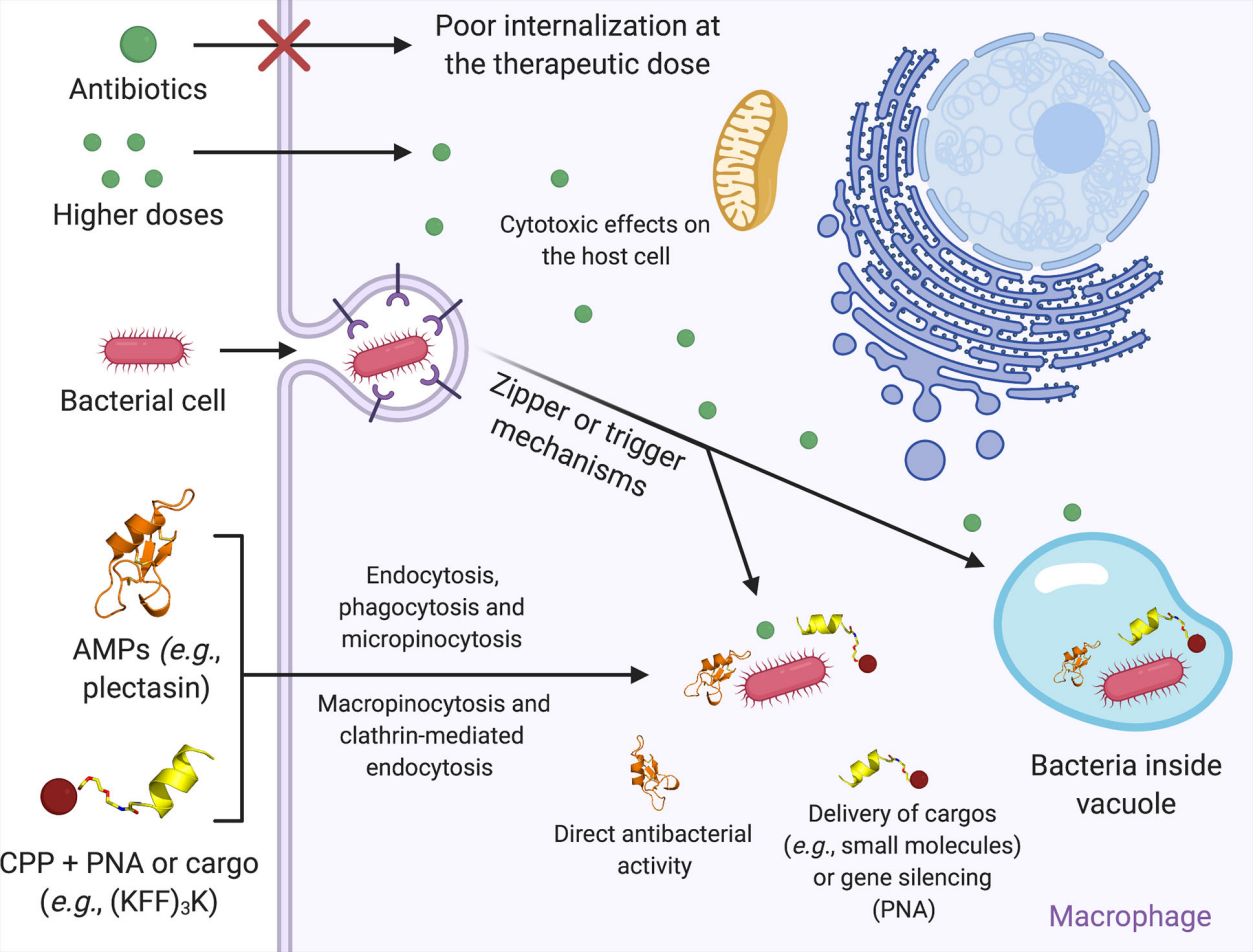
Schematic representation of the mechanism involved in bacteria, AMPs, and CPPs internalization into macrophages. Free-floating bacteria can invade the host cell by zipper or trigger mechanisms. Once inside the host cell, intracellular bacteria can occupy both the cytosol and vacuoles. At their therapeutic dose, conventional antibiotics usually present low permeability, rendering the intracellular bacterial infection treatment ineffective. By contrast, increasing antibiotics’ concentration can favor their permeabilization across the host cell membrane to treat the infection. However, as a consequence, it can also trigger cell toxicity. AMPs and CPPs have been used as alternative therapies. These peptide classes can translocate through the host cell plasma membrane *via* endocytosis, phagocytosis and macropinocytosis. In some cases, CPPs can also form transient toroidal pores or interact with membrane receptors. Once inside the cell, AMPs usually trigger direct antibacterial activities, whereas CPPs have been commonly used in conjunction with PNA and cargos aiming at essential bacterial genes silencing or direct antibacterial activities, respectively. Figure created with BioRender.com.

Based on their activities and structural profiles, new and promising active molecules have been designed; among them, we can cite AMPs with intracellular antibacterial activity (Crépin et al., 2020). In general, these peptides are constituted of five to 50 amino acid residues and manage to cross the host’s first barrier, the plasma membrane (Felício et al., 2017). Once internalized by endocytosis or macropinocytosis (**Figure 1**), they can present direct actions in vacuoles or cytosol, where the bacteria are found. Moreover, they can interfere with some internal signaling pathways *(e.g.*, maturation of IL-1β, and increased levels of TNF-α, IL-1β, and IL-10 to eliminate intracellular bacteria) (Wang et al., 2018). By interfering with the intracellular pathways, some specific mediators are increased, including those responsible for chemotactic activities, stimulation of the oxidative metabolism in phagocytes, increasing protein production in the acute inflammation phase and stimulating CD4 + and CD8 + cells (Trinchieri, 1997; O’Shea et al., 2002).

Cell-penetrating peptides (CPPs) are also considered intracellular antibacterial agents as they can penetrate bacterial cell membranes without seriously compromising their integrity. CPPs are small peptides that can autonomously translocate across plasma membranes and mediate transport of cargo molecules (Gomarasca et al., 2017). Charge molecules that can be coupled to CPPs include biological products, such as proteins, oligonucleotides, nanoparticles, and small molecule drugs (Hoffmann et al., 2018). The conjugation of CPP-charges can occur by a covalent and non-covalent bond (Hoffmann et al., 2018). The main feature of the plasma membrane is to protect the internal content of the cells. Therefore, the plasma membrane is a semipermeable barrier and essential for cell survival. The transport of compounds through the membrane occurs freely with small molecules or specific channels in larger molecules (Gomarasca et al., 2017; Ruseska and Zimmer, 2020). CPPs have a high permeability rate, cross the membrane of different cell types, present low cytotoxicity and do not activate the host’s immune response (Ruseska and Zimmer, 2020).

These peptides can either exert a direct antibacterial mechanism or perform the delivery of bioactive molecules, including other antimicrobial agents and PNAs (Reissmann, 2014). PNAs are synthetic DNA derivatives applied in hybridization-based microbial diagnostics. They are able to reduce the proliferation of various bacteria due to the antisense targeting of essential genes (Barkowsky et al., 2019). PNAs coupled to CPPs are able to support translocation over the cell membrane in bacteria (**Figure 1**) and, thus, increase antimicrobial efficiency (Barkowsky et al., 2019). The CPPs’ internalization mechanisms depend on two factors, including the target cell and the load coupled to the CPPs (when used in association with cargos) (Ruseska and Zimmer, 2020).

In this mini-review, we cover recent findings on the usage of AMPs and CPPs as promising intracellular antibacterial candidates, which act by interfering with cell-penetrating properties, and their suggested mechanism of action and conjugation with other antimicrobial agents.

## Intracellular Infections Caused by Multidrug-Resistant Bacteria

Bacteria are divided into two groups, including extracellular and intracellular bacteria. Extracellular bacteria have a free livelihood in environmental niches, whereas intracellular bacteria can infect a host cell and further replicate (McClure et al., 2017). Among the main obligate intracellular bacteria, we can mention *Chlamydia* spp., *Anaplasma* spp., *Ehrlichia* spp., *Rickettsia* spp., *Orientia* spp., and *Coxiella burnetii* (McClure et al., 2017; Otten et al., 2018), whereas facultative intracellular bacteria include *Salmonella* spp., *Francisella* spp., *Legionella pneumophila, Listeria monocytogenes*, and *Yersinia* spp (McClure et al., 2017; Otten et al., 2018).

Obligatory or facultative intracellular lifestyles are characteristic of bacterial evolution, enabling these microorganisms’ growth and replication in different niches (Otten et al., 2018). When bacteria start the cell internalization process, some of them can be detected by the host defense mechanisms, activating macrophage recruitment. However, bacteria that evade this process can also internalize macrophages, thus being protected from the host’s immune response and antibiotic action (Maurin and Raoult, 2001; Canton and Kima, 2012). Thus, intracellular bacteria reside directly in the mammalian cell-host cytoplasm or vacuoles, manipulating endocytic or secretory pathways to recruit necessary supplements to guarantee their replication (Loeuillet et al., 2006; Canton and Kima, 2012; Yeh et al., 2020). Different systems can facilitate the entry of intracellular bacteria in professional phagocytes, including type III secretion system (T3SS) and type IV secretion system (T4SS) (Coburn et al., 2007). These systems can translate specific proteins (effector proteins) to mediate pathways of the pathogen’s internalization. The purpose of bacterial effector proteins is to facilitate the entry of intracellular bacteria into the host cell, providing survival and replication in the cytoplasm or intracellular organelle (Ham et al., 2011; Yeh et al., 2020).

Intracellular bacteria can invade different cell types, including neutrophils, keratinocytes, and intestinal epithelial cells (Ham et al., 2011; Abu-Humaidan et al., 2018). The strategies used by intracellular bacteria to invade non-phagocytic host cells include the zipper and trigger mechanisms (**Figure 1**) (Ham et al., 2011). Bacteria that use the zipper mechanism express proteins on their membrane surfaces, which bind to the host cell’s plasma membrane receptors. The binding of surface proteins (bacteria and host cells) promotes pathogen-host cell interaction and triggers signaling cascades that reorganize the actin cytoskeleton, helping the bacteria to be internalized (Cossart and Sansonetti, 2004; Ham et al., 2011). By contrast, the trigger mechanism is characterized by the type III secretion system (Veiga and Cossart, 2006). The bacterium secretes effector proteins into the cytoplasm to modulate the actin cytoskeleton, polymerizing the actin, thus forming a pseudopod and allowing the bacteria’s entry into the cytoplasm (Veiga and Cossart, 2006; Ó Cróinín and Backert, 2012).

Another problem is that bacterial infections caused by intracellular pathogens are incredibly difficult to eradicate (Cossart and Sansonetti, 2004), as the antibiotics’ concentration internalized in host cells is lower than their minimum inhibitory concentration (MIC) (Harish and Menezes, 2015). Such disparities in antibiotic concentration could lead to further drug resistance. The antimicrobials currently used to treat intracellular pathogens are sulfonamides, quinolones, tetracyclines, and beta-lactams (Harish and Menezes, 2015; Bongers et al., 2019). It is suggested that sulfonamides are captured by host cells by active mechanisms and have a bacteriostatic effect on the intracellular pathogen. Quinolones enter and accumulate in subcellular compartments. Beta-lactams enters the cell by passive diffusion, interfering with the intracellular bacteria peptidoglycan synthesis. Finally, tetracyclines enter the host cells through organic cations active transport and interfere with bacterial protein synthesis (Bongers et al., 2019). Nevertheless, the recurrent choice of these antibiotics has become limited in clinical cases with resistant bacterial strains (Harish and Menezes, 2015).

The increase in resistance to multiple drugs reinforces the urgent need to develop new intracellularly active antibacterial agents capable of overcoming the low cellular permeability of the antimicrobials currently used (Weissman et al., 2016).

## Antimicrobial Peptides

Antimicrobial peptides (AMPs) are multifunctional molecules that have been largely isolated from various organisms, including vertebrate and invertebrate animals, plants, and bacteria (Porto et al., 2017). AMPs can be useful in eliminating bacteria, parasites, fungi, and viruses (Yan and Hancock, 2001). In addition, immunomodulatory and anticancer properties have also been reported (Hancock and Sahl, 2006). Therefore, the range of physicochemical properties and mechanisms of action attributed to AMPs make these molecules promising antibacterial drug candidates, which are more capable of acting on different biosynthetic pathways than conventional antibiotics reported as working against extracellular and intracellular bacteria (Gottschalk et al., 2015; Silva and Gomes, 2017).

Amongst multiple AMPs used for intracellular pathogens is plectasin (**Figure 1**). This peptide derived from the fungus *Pseudoplectania nigrella* shows defensin-like characteristics found in spiders, scorpions, dragonflies and mussels (Mygind et al., 2005). This peptide has been evaluated against methicillin-resistant *S. aureus* (MRSA) in its extra and intracellular modes of infection (Brinch et al., 2009; Xiong et al., 2011; Wang et al., 2018). Wild-type plectasin (NZ2000) has been used to treat monocytes (model THP-1) infected by MRSA, leading to a reduction of 1-log in the intracellular bacterial load (**Table 1**) (Brinch et al., 2009). This *in vitro* intracellular plectasin activity was more effective than vancomycin (Brinch et al., 2009). Moreover, the plectasin analogs NZ2114 and MP1102 were evaluated against three *S. aureus* strains (methicillin-susceptible and –resistant strains, and a high virulence strain) internalized in RAW 264.7 cells (**Table 1**). Compared to their parent peptide (plectasin), both analogs effectively reduced the intracellular bacterial load (Wang et al., 2018). Other defensin-like AMPs, including HNP-1 (α-human defensins) and RC-1 (humanized θ-defensin, retrocyclin – 1), were tested against intracellular *Listeria monocytogenes* (Arnett et al., 2011). All these peptides caused a dose-dependent inhibition of bacterial proliferation, with more promising results for RC-1 (**Table 1**) (Arnett et al., 2011).

**Table 1:** Antimicrobial peptides (AMPs) and cell-penetrating peptides (CPPs) activity against intracellular pathogenic bacteria.

Peptide	Intracellular Bacterial	Minimal Inhibitory Concentration (µg mL^-1^)	Mode of Penetration into Infected Cell	Test Development Phase	Reference
**AMPs**					
**Wild-type plectasin (NZ2000)**	*S. aureus* (MSSA) *S. aureus (*ATCC 25923)	8 128	nd	*In vitro* and *in vivo*	(Brinch et al., 2009; Lacoma et al., 2017)
**Plectasin analogs** **MP1102**	*S. aureus* (MSSA) *S. aureus* (MRSA)	0.15–31 1.25–250	nd	*In vitro* and *in vivo*	(Lacoma et al., 2017; Wang et al., 2018)
**Plectasin analogs** **NZ2114**	*S. aureus* (MSSA) *S. aureus* (MRSA) Virulent *S. aureus* CVCC546	0.3–60 1.25–250 100–200	Involved clathrin-mediated endocytosis and micropinocytosis	*In vitro* and *in vivo*	(Lacoma et al., 2017; Wang et al., 2018)
**Humanized θ-defensin retrocyclin RC-1**	*Listeria monocytogenes*	1	Enter target cells *via* phagocytosis or induced endocytosis	*In vitro*	(Arnett et al., 2011; Witter et al., 2016; Lacoma et al., 2017)
**α-defensin HNP-1**	*Listeria monocytogenes*	20	Enter target cells *via* phagocytosis or induced endocytosis	*In vitro*	(Arnett et al., 2011; Witter et al., 2016; Lacoma et al., 2017)
**Esculentin-1a (1–21)**	*Pseudomonas aeruginosa*	11–33	nd	*In vitro*	(Cappiello et al., 2016)
**Diastereomer analog, Esc (1–21)-1c**	*Pseudomonas aeruginosa*	11–33	nd	*In vitro*	(Cappiello et al., 2016)
**Insect defensin-like peptide DLP4**	*S. aureus* (MRSA)	20	nd	*In vitro*	(Lacoma et al., 2017; Li et al., 2017)
**DLP2 analog DLP4**	*S. aureus* (MRSA)	10	nd	*In vitro*	(Li et al., 2017)
**WR12**	*S. aureus* (MRSA) *S. aureus* (MSSA)	16.5 33	nd	*In vitro* and *in vivo*	(Mohamed et al., 2016)
**D-IK8**	*S. aureus* (MRSA) *S. aureus* (MSSA)	16.5 33.5	nd	*In vitro* and *in vivo*	(Mohamed et al., 2016)
**BSN-37**	*Salmonella enterica* serovar *Typhimurium*	400	nd	*In vitro*	(Yang et al., 2020)
**CPPs**					
**Tat11 - conjugate AMP-N2** **bLFcin6 - conjugate AMP-N2**	*Salmonella enterica* serovar *Typhimurium*	205 355	energy-dependent macropinocytosis and clathrin-mediated endocytosis pathways	*In vitro*	(Li et al., 2018)
**PNA-CPP, PRXR**	*Listeria monocytogenes*	11	nd	*In vitro* and *in vivo*	(Abushahba et al., 2016)
**Tat-gentamicin**	*E. coli* K1	600	nd	*In vitro*	(Gomarasca et al., 2017)
**P14KanS**	*Mycobacterium tuberculosis* *Salmonella*	33	nd	*In vitro* and *in vivo*	(Brezden et al., 2016)

Amphibian-derived peptides (frog skin), including esculentin-1a (Trinchieri, 1997; O’Shea et al., 2002; Monack et al., 2004; Proctor et al., 2006; Splith and Neundorf, 2011; Reissmann, 2014; Lehar et al., 2015; Mitchell et al., 2016; Cornejo et al., 2017; Le et al., 2017; Felício et al., 2017; Gomarasca et al., 2017; McClure et al., 2017; Kumar et al., 2018; Wang et al., 2018; Hoffmann et al., 2018; Otten et al., 2018; Barkowsky et al., 2019; Yount et al., 2019; Crépin et al., 2020; Ruseska and Zimmer, 2020) (Islas-Rodrìguez et al., 2009; Luca et al., 2013) and its diastereomer analog, named Esc (Trinchieri, 1997; O’Shea et al., 2002; Monack et al., 2004; Proctor et al., 2006; Splith and Neundorf, 2011; Reissmann, 2014; Lehar et al., 2015; Mitchell et al., 2016; Cornejo et al., 2017; Felício et al., 2017; Gomarasca et al., 2017; Le et al., 2017; McClure et al., 2017; Kumar et al., 2018; Hoffmann et al., 2018; Otten et al., 2018; Wang et al., 2018; Barkowsky et al., 2019; Yount et al., 2019; Crépin et al., 2020; Ruseska and Zimmer, 2020)-1c, have been tested for their intracellular antibacterial potential on two bronchial cell types infected with *Pseudomonas aeruginosa* (**Table 1**) (Cappiello et al., 2016). At 5 μM, both peptides caused 15 to 20% intracellular bacterial death within 1 h after treatment in bronchial cells. In contrast, when tested in ΔF508 bronchial cells, at 5 μM, the esculentin-1a (Trinchieri, 1997; O’Shea et al., 2002; Monack et al., 2004; Proctor et al., 2006; Splith and Neundorf, 2011; Reissmann, 2014; Lehar et al., 2015; Mitchell et al., 2016; Cornejo et al., 2017; Le et al., 2017; Felício et al., 2017; Gomarasca et al., 2017; McClure et al., 2017; Kumar et al., 2018; Hoffmann et al., 2018; Otten et al., 2018; Wang et al., 2018; Yount et al., 2019; Barkowsky et al., 2019; Crépin et al., 2020; Ruseska and Zimmer, 2020) peptide reduced 40% of the intracellular bacteria load, whereas its diastereomer analog caused 60% bacterial reduction (Cappiello et al., 2016).

Insects have essential AMPs from their organism’s innate system, a rich source to be prospected (Li et al., 2018). The DLP4 peptide, isolated from the hemolymph of *Hermetia illucens*, showed potent antibacterial activity against Gram-positive and negative. In an assay using MRSA ATCC43300 in intracellular form with RAW 264.7 cells; DLP4 peptides and their analog DLP2 significantly reduced bacterial growth *in vitro* (DLP2 - 1.89 -log and DLP4 - 1.34 -log) (**Table 1**) (Li et al., 2018).

A mammalian peptide, denominated BSN-37 {a truncated N-terminal fragment [Bac5 (Trinchieri, 1997; Maurin and Raoult, 2001; Yan and Hancock, 2001; O’Shea et al., 2002; Cossart and Sansonetti, 2004; Monack et al., 2004; Hancock and Sahl, 2006; Loeuillet et al., 2006; Proctor et al., 2006; Veiga and Cossart, 2006; Coburn et al., 2007; Ham et al., 2011; Splith and Neundorf, 2011; Canton and Kima, 2012; Ó Cróinín and Backert, 2012; Reissmann, 2014; Gottschalk et al., 2015; Harish and Menezes, 2015; Lehar et al., 2015; Mitchell et al., 2016; Weissman et al., 2016; Felício et al., 2017; Gomarasca et al., 2017; Le et al., 2017; McClure et al., 2017; Porto et al., 2017; Abu-Humaidan et al., 2018; Hoffmann et al., 2018; Kumar et al., 2018; Wang et al., 2018; Otten et al., 2018; Barkowsky et al., 2019; Bongers et al., 2019; Yount et al., 2019; Crépin et al., 2020; Ruseska and Zimmer, 2020; Yeh et al., 2020)] of Bac5}, has been evaluated against intracellular *Salmonella enterica* serovar *Typhimurium* (*S. Typhimurium*) bacteria (Yang et al., 2020). For the intracellular experiment, Madin-Darby canine kidney cells (MDCK) were used and the peptide BSN-37 antibacterial effect was evaluated. It was observed that BSN-37 reduced the amount of intracellular S*. Typhimurium*, suggesting that this peptide can enter the host cell and kill bacteria without harming the cellular viability of MDCK (**Table 1**) (Yang et al., 2020).

Rational design is a modern approach used to improve existing peptide structures for a desired biological activity. The peptide WR12 (RWWRWWRRWWRR) is a *de novo* designed short synthetic peptide, composed exclusively of arginine and tryptophan amino acid residues. D-IK8 (irikirik) is a short synthetic peptide composed only of eight D-amino acids (Mohamed et al., 2016). These peptides were tested for their intracellular antibacterial activity on human keratinocytes infected by MRSA and methicillin-sensitive *S. aureus* (MSSA) (Mohamed et al., 2016). The D-IK8 peptide reduced the intracellular bacterial load of MRSA and MSSA by 96%, at a concentration of 16 µM, whereas at this same concentration, the peptide WR12 reduced MRSA and MSSA loads by 40% (**Table 1**) (Mohamed et al., 2016).

Many AMPs have remarkable activity against bacteria, but the minority of AMPs manage to reach intracellular bacteria *via* nonlytic mechanisms to the host cell (Splith and Neundorf, 2011; Yang et al., 2020). An alternative is the CPPs, which can present intracellular antimicrobial activity or can be coupled to cargos with activity against intracellular bacteria (Splith and Neundorf, 2011).

## Cell-Penetrating Peptides

Frankel and Pabo were the first researchers to discover a peptide capable of translocating to the nucleus of cells, known as the TAT peptide (transactivating transcription protein), encoded by human immunodeficiency virus type 1 (HIV-1) (Frankel and Pabo, 1988). Later, consecutive discoveries were made and, currently, more than 1,700 CPPs have been registered in an online database (http://crdd.osdd.net/raghava/cppsite/).

The mechanisms of CPP’s penetration into cells can occur by more than one pathway. In general, the internalization of CPPs is closely related to the molecule conjugated to it (cargo), its concentration and the target cell type (Koren and Torchilin, 2012). Authors suggest that the mechanisms may include endocytosis followed by endosomal escape, which can be differentiated in many ways, including macropinocytosis, clathrin-mediated endocytosis, lipid raft-mediated endocytosis or caveola-mediated endocytosis, or *via* direct membrane penetration, for example, by transient toroidal pores or micelles formation (**Figure 1**) (Richard et al., 2003; Madani et al., 2011; Wang et al., 2014; Rizzuti et al., 2015).

AMP-N2; a marine NZ17074 peptide analog isolated from the invertebrate lugworm *Arenicola marina*, were covalently conjugated with two CPPs. CPP-N2 conjugates are composed of a target peptide-N2 linker and CPPs Tat11 (trans-activator of transcription-Tat11 peptide) or bLFcin6 (bovine lactoferricin), and both were tested against intracellular *S. Typhimurium* (Li et al., 2018). This bacterium was internalized into RAW 264.7 cells and treated with 10, 20, and 50 µM of T11N2 and 50 and 100 µM of B6N2. T11N2 reduced the intracellular bacterial load by 3.26-log at 50 µM, whereas B6N2 reduced it by 2.1-log at 100 µM (**Table 1**) (Li et al., 2018). The authors suggest that the internalization of conjugated CPPs could occur through energy-dependent macropinocytosis and clathrin-mediated endocytosis pathways (Li et al., 2018).

CPPs have also been used to carry PNAs, aiming at silencing bacteria’s essential genes (Bai et al., 2012). For a PNA to enter the cell, it is necessary to attach it to a CPP (Bai et al., 2012). For instance, the essential RNA polymerase α-subunit (encoded by the rpoA gene) in the intracellular pathogen *Listeria monocytogenes* has been a target for PNA-CPP therapies. Abushahba et al. evaluated five different CPPs [antennapedia, TAT, (RXR) 4XB, and (RFR) 4XB] coupled with a PNA targeting the gene cited above (Abushahba et al., 2016). *Listeria monocytogenes* was internalized in J774A.1 cells and treated with the PNA-CPP candidates at 2, 4, and 8 μM. Among the tested PNA-CPPs, PRXR showed a higher reduction in the bacterial load (Log _10_ 1.78), at a 2 μM (**Table 1**). However, the cell permeabilization mechanism of action triggered by this CPP is still unknown (Abushahba et al., 2016). Interestingly, studies have reported that encapsulated intracellular bacteria (*e.g.*, *S. Typhimurium* LT2) are commonly more difficult to treat with PNA-CPP therapies (Cornejo et al., 2017). As an alternative, the electroporation-based delivery of CPP-PNA conjugates has been proposed, which has led to increased bioavailability, as this strategy does not involve the conjugate endocytosis (Cornejo et al., 2017).

Another applicability of CPPs includes the transport of antibiotics into the host cell, as these antimicrobials can be useful against intracellular bacteria, but have little permeability. To evaluate whether gentamicin (GM) would target intracellular bacteria, studies have proposed combining GM with CPPs (Gomarasca et al., 2017). CPPs, including α1H (KSKTEYYNAWAVWERNAPC), α2H (GNGEQREM AVSRLRDCLDRQA), and TAT peptide have been used in this regard (Gomarasca et al., 2017). In a study by Gomarasca et al (Trinchieri, 1997)., human brain microvascular cells (HBMEC) were infected with *E. coli* K1 and treated with 600 µg mL^-1^ of the three gentamicin-CPP conjugates, including α1H-gentamicin; α2H-gentamicin and Tat-gentamicin (Gomarasca et al., 2017). The best result was observed by treating the cells with Tat-gentamicin, which led to a 6-log reduction in bacterial load (**Table 1**) (Gomarasca et al., 2017). Transmission electron microscopy studies indicate that eukaryotic cells’ surface integrity was maintained. Additionally, it was observed that the intracellular bacteria remain in endosomal compartments or vacuoles after treatment with Tat-gentamicin, ultimately losing their cell-wall integrity (Gomarasca et al., 2017). Similarly, the development of a cleavable kanamycin-CPP conjugate has been reported (Brezden et al., 2016). This conjugate (P14KanS) containing the disulfide linkage will be broken to release kanamycin when cleaved in the intracellular reduction environment. P14KanS presents high cell penetration potential and significant antibacterial potential against *Mycobacterium tuberculosis* and *Salmonella*, both *in vitro* and *in vivo* (**Table 1**) (Brezden et al., 2016).

## Conclusion

Intracellular bacterial infections pose an additional challenge compared to extracellular infections, as the antibacterial agent of choice has to cross the host’s plasma membrane without harming the cell to eliminate the intracellular pathogen. High doses of conventional antibiotics are usually needed to successfully combat intracellular infections, considering their low cell-penetrating potential. As a consequence of these higher doses, host cell viability is often compromised. As alternative therapies, here we summarize recent findings on the usage of AMPs and CPPs for treating intracellular bacterial infections. As described, these classes of antimicrobials have shown the ability to reach the intracellular bacteria inside the host cell either to exert direct antibacterial activities or deliver cargo molecules with antibacterial potential. Moreover, many studies have reported the non-toxic effects of these peptides on the host cells, rendering them attractive drug candidates for countering intracellular infections.

## Author Contributions

DB and MC contributed equally. MC and OF corrected the manuscript. All authors contributed to the article and approved the submitted version.

## Funding

This work was supported by Coordenação de Aperfeiçoamento de Pessoal de Nível Superior (CAPES), Conselho Nacional de Pesquisa e Desenvolvimento (CNPq), Fundação de Apoio à Pesquisa do Distrito Federal (FAPDF), and Fundação de Apoio ao Desenvolvimento do Ensino, Ciência e Tecnologia do Estado de Mato Grosso do Sul (FUNDECT).

## Conflict of Interest

The authors declare that the research was conducted in the absence of any commercial or financial relationships that could be construed as a potential conflict of interest.
